# How can health systems research reach the worst-off? A conceptual exploration

**DOI:** 10.1186/s12913-016-1868-6

**Published:** 2016-11-15

**Authors:** Bridget Pratt, Adnan A. Hyder

**Affiliations:** 1 Nossal Institute for Global Health, School of Population and Global Health, University of Melbourne, 161 Barry Street Carlton, VIC 3053 Australia; 2 Department of International Health, Johns Hopkins Bloomberg School of Public Health, 615 N Wolfe St, Baltimore, MD 21205 USA; 3 Johns Hopkins Berman Institute of Bioethics, 1809 Ashland Avenue, Baltimore, MD 21205 USA

## Abstract

**Background:**

Health systems research is increasingly being conducted in low and middle-income countries (LMICs). Such research should aim to reduce health disparities between and within countries as a matter of global justice. For such research to do so, ethical guidance that is consistent with egalitarian theories of social justice proposes it ought to (amongst other things) focus on worst-off countries and research populations. Yet who constitutes the worst-off is not well-defined.

**Methods and Results:**

By applying existing work on disadvantage from political philosophy, the paper demonstrates that (at least) two options exist for how to define the worst-off upon whom equity-oriented health systems research should focus: those who are worst-off in terms of health or those who are systematically disadvantaged. The paper describes in detail how both concepts can be understood and what metrics can be relied upon to identify worst-off countries and research populations at the sub-national level (groups, communities). To demonstrate how each can be used, the paper considers two real-world cases of health systems research and whether their choice of country (Uganda, India) and research population in 2011 would have been classified as amongst the worst-off according to the proposed concepts.

**Conclusions:**

The two proposed concepts can classify different countries and sub-national populations as worst-off. It is recommended that health researchers (or other actors) should use the concept that best reflects their moral commitments—namely, to perform research focused on reducing health inequalities or systematic disadvantage more broadly. If addressing the latter, it is recommended that they rely on the multidimensional poverty approach rather than the income approach to identify worst-off populations.

## Background

Health systems research (HSR) is increasingly performed in low and middle-income countries (LMICs) and is largely externally-funded, with growing investments coming from bilateral aid agencies, philanthropies, and national research bodies in high-income countries [1]. Conceptual work in bioethics has argued that, as a matter of global justice, HSR should generate knowledge to enhance service delivery and affordability for disadvantaged groups [2]. This work is part of a broadened bioethics research agenda that explores how international research can promote justice in global health and that considers the ethics of HSR [3–5]. It is consistent with statements made by the World Health Organization and at global ministerial summits on health research, which identify HSR as an essential means of reducing health disparities between and within countries [6]. In practice, many HSR studies, especially in LMICs, are conducted with the primary aim of enhancing health system performance for disadvantaged groups or communities [3]. This is particularly true for a rising number of studies that emphasise community participation and empowerment in health service planning, delivery, and evaluation. Such an approach is informed by growing consensus on the value of community participation in improving health systems and outcomes, and the idea that community capacities can be harnessed to generate these improvements [7–9]. In this paper, health systems are understood to encompass public health and health care systems.

What features are necessary for HSR projects in LMICs to promote health equity has also been explored by bioethicists. Consensus exists amongst broadly egalitarian social justice theories that the most urgent task is to identify the worst-off and take appropriate steps so that their position can be improved [10]. There is, in effect, support across theories of social justice for the guidance articulated in a recently proposed ethical framework called ‘research for health justice’, which links HSR in LMICs to the promotion of global justice [2]. ‘Research for health justice’ affirms that HSR in LMICs should (amongst other things) focus on worst-off populations and their needs. This does not mean other factors cannot be considered when selecting amongst worst-off populations such as feasibility or existing relationships. (We further note the very use of such terms, however benignly intended, risks offending those so labelled; the substitution of terms that connote agency and personhood would in itself be more inclusive). The framework also provides guidance on what research questions should be selected, what research capacity strengthening should be performed, and what post-study benefits ought to be provided and to whom [2].

Since HSR in LMICs is generally performed with sub-national populations in particular countries, for external researchers from high-income countries, upholding ‘research for health justice’ entails first identifying worst-off countries in which to work and then identifying worst-off sub-national populations within them upon whom to focus needed research. For researchers from LMICs, who are already based in-country, it would entail identifying worst-off sub-national populations. These sub-national populations might be communities (geographically-based or otherwise), political jurisdictions (districts, sub-districts), or groups with shared characteristics or experiences of social arrangements (policies, rules, norms) that generate disadvantage. Worst-off sub-populations are not limited to citizens of countries. Additionally, while health systems researchers are obligated to focus their studies on worst-off populations, they are not the only actors who should or can identify such populations. For instance, LMIC policymakers can designate priority populations within their countries in order to guide researchers’ selection of sub-national populations.

Yet *who* constitutes the worst-off is not well-defined. Little, if any, work has been done to specify how to identify worst-off countries or research populations at the sub-national level. It cannot be assumed simply conducting HSR in a LMIC is sufficient for such research to reach the worst-off and concentrate on their needs. All LMIC populations may not be considered “worst-off”. Conceptual work is needed to *operationalise* what selecting worst-off countries and research populations entails. Such guidance will help facilitate the ethical conduct of HSR. It will support researchers to put ‘research for health justice’ into practice and can usefully inform their design of HSR projects seeking to unlock community capabilities. Where HSR is targeted at worst-off groups and communities within LMICs, it will be better placed to empower them. Worst-off groups and communities’ active participation in their health systems would be visibly enhanced, promoting greater system responsiveness to their needs.

In this paper, we adopt the following approach to help clarify how to select worst-off countries and research populations: 1) develop a broad definition of worst-off, 2) describe the features or proxies that can be used to identify national and sub-national populations that meet the definition, and 3) identify metrics. First, existing concepts of disadvantage from political philosophy are applied to the research context. We propose that (at least) two options exist for how to define the worst-off upon whom HSR in LMICs should focus: those who are least advantaged in terms of health and those who are least advantaged in terms of multiple dimensions of well-being (systematically disadvantaged). Second, detailed descriptions of how each broad definition can be understood at the national and sub-national levels are provided. To do so, we draw on existing work from bioethics and philosophy that identifies proxies for systematic disadvantage and features of being worst-off in health. Finally, the proxies and features are linked to possible metrics that can be used to identify worst-off countries and sub-national populations.

To demonstrate how to employ the two concepts to identify worst-off populations in practice, the paper uses them to classify two real-world HSR projects’ (see “Cases of international health research projects,” below) choice of country and research population. Doing so also enables assessment of whether applying the concepts is likely to result in different classifications of the same populations; the conceptual differences between them may have implications for who is identified as a worst-off country and research population. The particular cases were chosen because they constitute HSR projects that aim to harness community assets to reduce unequal access to health services in LMICs. Although the cases had equity objectives, it was unclear whether the populations they focused on, which varied considerably from one another, were worst-off at the time of their selection in 2011. For that reason, it was considered useful to classify the cases’ choices of country and research population. (It should be noted that the data used to do so reflects what was available in 2011 and/or is from 2011).

### Cases of international health research projects

#### Case 1

A HSR study was undertaken in three rural districts (Kamuli, Kibuku, and Pallisa) in eastern Uganda from 2011 to 2016. The aim of the study was to determine whether or not the utilization of maternal and child health services could be increased in the three districts by engaging their communities and health service providers to enhance awareness about maternal and newborn care, improve financial preparedness of households for delivery, and link families with transport to health centres.

#### Case 2

A HSR study was undertaken in the Sundarbans in the Indian state of West Bengal from 2011 to 2016. The aim of the study was to assess the effectiveness of an intervention designed to lead West Bengal policy actors to allocate more resources towards addressing the service delivery gap in the Sundarbans.

## Two definitions of worst-off

Political philosophy offers a rich conceptual resource on the topic of disadvantage that has yet to be applied to the research context. Recent scholarship has explored the meaning of disadvantage and that body of work suggests that the concept of worst-off can be interpreted in two main ways. As noted by Wolff and de-Shalit, assuming that well-being is pluralistic (i.e. encompassing multiple dimensions such as health, security, self-determination), it is not clear whether we should pay attention to the claims of the least advantaged overall or the least advantaged with respect to a particular dimension of functioning, even where the group is not doing particularly badly overall [10]. This question can be re-framed as whether the worst-off should be defined as those who are worst-off in health terms or those who are worst-off across multiple dimensions of well-being that include health?

Theories of social justice that address disadvantage and health collectively lend support to both concepts of the worst-off. These, most recently, include the work of Madison Powers and Ruth Faden, Jonathan Wolff and Avner de-Shalit, Martha Nussbaum, Jennifer Prah Ruger, and Sridhar Venkatapuram [10–14]. (The latter three theorists’ work extend Sen’s capability approach). Powers and Faden’s theory of social justice considers the worst-off to be those who are *systematically disadvantaged*—namely, those who fall below a level of sufficiency on multiple dimensions of well-being. Their theory identifies six essential dimensions of well-being—health, reasoning, self-determination, attachment, personal security, and respect—and contends that a life “substantially lacking in in any one [of those dimensions] is a life seriously deficient in what is reasonable for anyone to want” ([11], p. 6). However, the populations whose deficits in health are most “morally urgent” to address are those who experience deficits on multiple dimensions that are caused by multiple social determinants ([11], p. 87). These deficits comprise sizeable deficits from sufficiency rather than the absolute largest deficits. Populations whose deficits in health are part of a systematic pattern of disadvantage are the worst-off upon whom the field of public health (including public health research) should concentrate [11].

Powers and Faden’s work further emphasises that disadvantage is not only characterised by deprivations in core dimensions of well-being but also created and entrenched through institutional arrangements and social practices. Social arrangements or structures (rules, policies, norms) are organised to favour some over others, thereby making it much harder for certain groups and communities to achieve well-being [11]. Mechanisms that create and sustain systematic disadvantage are identified as comprising: 1) forms of oppression and subordination such as colonialism, racism, gender bias, and stigmatization of members of groups and 2) concentrations of power, resources, and privileged social standing that result in the structuring of social, economic, and political arrangements to benefit dominant groups and reinforce existing inequalities [11].

Wolff and de-Shalit’s work on disadvantage also emphasises its plurality, arguing that deficits on certain categories of functioning such as life; health; bodily integrity; affiliation; control over one’s environment; and sense, imagination, and thought are particularly important [10]. These categories overlap, to some extent, with Powers and Faden’s dimensions of well-being. Wolff and de-Shalit further introduce the concept of *corrosive disadvantage*, which has been endorsed by Nussbaum (albeit with some caveats) and refers to people experiencing large shortfalls and/or insecurity on a cluster of these high-priority functionings [10, 12]. Shortfalls refer to actual low achievement of the functionings. Insecurity means having a low probability of sustaining an achieved functioning [10]. (Nussbaum endorses this concept in terms of capabilities [12]). The worst-off are those individuals who experience corrosive disadvantages, which, in turn, suggests that HSR should focus on those who experience sizeable shortfalls and/or insecurity on a cluster of functionings that include health.

In contrast, Ruger’s health capability paradigm considers those who experience large shortfalls in their health capabilities from the optimal level achieved worldwide to be the worst-off populations upon whom HSR should focus [2]. The optimal level of health refers to the highest level of population health achieved worldwide in terms of morbidity and mortality indicators. HSR should address the health needs of those who experience sizeable shortfalls from that level, irrespective of whether they experience shortfalls on other basic capabilities. Here, the worst-off are understood as those who are substantially badly off rather than as those who fall the absolute farthest from the optimal level of health.

Critiques of both concepts of worst-off have been articulated and relate to their philosophical justifications, commensurability, and/or exclusion of non-basic dimensions of well-being [15, 16]. However, we do not find these critiques render either of the concepts too deficient or implausible to explore further. We have also chosen to define the worst-off in terms of functionings, as do Powers, Faden, Wolff and de-Shalit, because capabilities cannot be as easily observed and this can create severe difficulties for operationalisation [10]. The remainder of the paper describes how the two concepts might be further understood and applied at the national and sub-national levels.

Prior to doing so, it is important to note that the body of work on disadvantage from political philosophy, like capability theory in general, considers the moral claims of individuals to be primary [17]. This theoretical work largely ignores or rejects the ways that capabilities or functionings at the community level are being articulated in practice [17]. By drawing on such work, the proposed concepts each identify the worst-off in terms of a lack of (aggregate) *individual* health and well-being rather than in terms of a lack of *community* health and well-being.

Community capabilities have been defined as characteristics that can foster their ability to identify, mobilise, and address problems in order to improve their health and well-being. These capabilities include resources (physical, financial, human), leadership, participatory decision-making, social and organisational networks, social cohesion, and collective efficacy [9, 18–20]. While acknowledging that the two proposed concepts of worst-off give primacy to the moral claims of individuals, but bearing work on community capability in mind, the paper *will* identify how consideration of community level characteristics can be incorporated into the two concepts’ application at the sub-national level. A lack of certain community characteristics can be used to help identify sub-national populations that are worst-off in terms of individuals’ health or well-being.

## Results

### Worst-off in terms of health

#### National level

Where the worst-off are defined solely in terms of health, external researchers from high-income countries ought to perform HSR in countries with the worst health in order to advance global health justice [2]. But what does it mean for countries to have the worst health? The paper draws attention to four features that are usefully considered to make such an assessment in regards to a given country: its level of health achievement, its level of health security, the length of time it has experienced poor achievement and/or insecurity, and its level of within-country health inequality (Table 1). These factors are drawn from the bioethics literature, which endorses them as key considerations when identifying the worst-off in health. Each is described below.

**Table 1:** Summary of features, proxies, and metrics to identify the worst-off

Worst off in health		Systematic disadvantage	
National level features or proxies	Possible metrics	National level features or proxies	Possible metrics
Low health achievement	Life expectancy, infant mortality rate, maternal mortality rate, other health indicators	Poverty; Domination	Gross domestic product data, Gross national product data, Multidimensional Poverty Index data
Low health security	Frequency of droughts, storms, flooding; Ranking on Fragile State Index		
Long duration	Performance on health indicators for 5-10+ years		
High within-country health inequality	Health and health system indicators—life expectancy, infant mortality rate, maternal mortality rate, access to particular health services—by gender, income, caste, education, geography, etc.		
Sub-national level features or proxies	Possible metrics	Sub-national level features or proxies	Possible metrics
Individual or community characteristics associated with poor health and/or social arrangements that create or entrench poor health	Substantial gap between health and health system indicators for sub-national population versus relevant comparator sub-national population shown by, for example, Lorenz curve, Concentration curve and index, and/or Slope and relative indices of inequality	Poverty; Domination; Lack of community capability	Below the poverty line data, Multidimensional Poverty Index data

*Achievement* refers to the level of population health attained by a given country. Countries that are worst-off would comprise those that perform substantially worse on morbidity and mortality indicators relative to a decent or optimal level of health [13]. For example, worst-off countries would exhibit a substantial shortfall in terms of their life expectancies, infant mortality rates, and maternal mortality rates relative to the best-performing countries. Their performance, for example, might place them in the bottom third of countries worldwide.

Nussbaum, Wolff and de-Shalit argue that disadvantage consists of having a low level of not only health achievement but also *health* (or capability) *security* [10, 12]. Countries with insecure health are those countries with a low prospect of sustaining their achieved level of population health. Wolff and de-Shalit propose that this would, for instance, refer to countries that are particularly prone to the effects of climate change or are conflict-affected [10]. (However, they acknowledge other proxies for health insecurity exist beyond these). Where climate change is considered, states that are worst-off in terms of their health include Bangladesh, Malawi, Vietnam, South Sudan, Sudan, and the Philippines [21]. Where being conflict-affected is considered, the worst-off again include Sudan and South Sudan as well as Somalia, the Central African Republic, Yemen, Syria, and Chad. Each of these states was identified as being a very high risk by the 2016 Fragile States Index [22].

Another factor to consider is whether countries are worst-off in terms of health achievement and security right now or whether the deficits they currently experience have been sustained for a considerable *length of time* (e.g. 5–10 years) [23]. It has been argued that individuals who have experienced their current deficits in health for a prolonged period are worse-off than individuals who have only just begun to experience such deficits [15]. In today’s world, this distinction may not be so important because numerous low-income countries have had sustained health deficits due to the effects of colonial and autocratic regimes.

A final consideration when determining which countries have the worst health is *within-country inequality*. The existence of unequal heath achievements within countries has been identified as a core aspect of health inequity [24, 25]. The distribution of health within countries matters when selecting LMICs in which to work. For example, if two countries have a life expectancy of 50 years but Country A has little inequality (most people have a life expectancy of 50 years) and Country B has a lot of inequality (75 % of the population has a life expectancy of 40 and 25 % have a life expectancy of 80 years), is Country B worse-off than Country A because a large proportion of its population have a life expectancy of 40 years? Where countries have high health inequality, they will contain populations at the sub-national level that are worse-off relative to populations at the sub-national level of countries with similar overall health outcomes but little in-country health inequality. Thus, while using aggregate health data to identify the worst-off will focus HSR in ways with high potential to reduce *between* country health inequalities, relying on such data alone may target HSR in ways that do not maximise its potential to reduce *within* country health inequalities.

Given these considerations, the worst-off in terms of health at the national level are interpreted to mean countries with all or some of the four aforementioned features. This would include countries with high levels of wealth and poor health such as Angola and Gabon, but the priority given to these countries would depend on how many of the four features they exhibit. Those countries that exhibit all four characteristics could be viewed as being of highest priority, followed by those that exhibit three characteristics, and so on. That countries exhibiting only one of the criteria are considered worst-off can potentially justify conducting HSR in countries with only either low health security or high health inequality. If a country’s population experiences a moderate health shortfall *and* a lot of health inequality such as Brazil or China, this likely means that it will have sub-national populations that have substantial health shortfalls and are, therefore, worst-off in terms of their health. If a country’s population exhibits a moderate health shortfall and low health security such as Vietnam (due to climate change), its population is likely to fall substantially below a decent or optimal level of health in the future. Research in these countries would be ethically permissible but of lesser priority than research in countries meeting multiple criteria.

To further demonstrate the application of this definition of worst-off, the countries selected to be the focus of the HSR cases (“Cases of international health research projects,” above) are classified. Uganda and India’s health achievements in terms of, for example, life expectancies, maternal mortality rates, and infant mortality rates placed them within the bottom 10-20 % and 30 % of countries worldwide respectively in 2011 (Table 2). This was compounded by the insecurity of these health achievements in the northern region of Uganda, which has faced cycles of violence and conflict for the past two decades [26]. India is particularly prone to the effects of climate change; it was amongst the 12 countries identified by the World Bank as being at *highest* risk for droughts, floods, and growing uncertainty in agriculture [21].

**Table 2:** Health achievement in India and Uganda compared to the optimal level achieved worldwide

	India	Uganda	Highest/lowest level achieved worldwide
Life expectancy	66.8 years	53.2 years	89.7 years
Ranking for life expectancy (of 221 countries)	160^th^	203^rd^	1^st^
Infant mortality rate	48 per 1,000 live births	62 per 1,000 live births	2 per 1,000 live births
Ranking for infant mortality rate (of 226 countries)	175^th^	197^th^	1^st^
Maternal mortality rate	200 per 100,000 live births	310 per 100,000 live births	2 per 100,000 live births
Ranking for maternal mortality rate (of 184 countries)	129^th^	147^th^	1^st^

Source: [46]

Both countries also faced substantial in-country health inequalities in 2011. Disparities in life expectancy, infant and under five mortality rates, and access to health services were experienced by women, the poor, and those living in rural areas within India and Uganda (Table 3). Life expectancy in Uganda ranged from 30 years to 60 years between districts [27] and from 56 years to 74 years between Indian states. There was a marked variation in the distribution of health within India along dimensions such as gender, caste, wealth, education, and geography [28]. Thus, both countries exhibited multiple features associated with being worst-off in terms of their health.

**Table 3:** Available data on health indicators for India and Uganda by state/district, income level, and urban–rural classification

Health indicator	Country	Demographic trait			
		Richest 20 %	Poorest 20 %	Urban	Rural
Under 5 mortality rate	India	34 deaths per 1,000 live births	101 deaths per 1,000 live births	52 deaths per 1,000 live births	82 deaths per 1,000 live births
	Uganda	108 deaths per 1,000 live births	172 deaths per 1,000 live births	115 deaths per 1,000 live births	147 deaths per 1,000 live births
Infant mortality rate	India	34 deaths per 1,000 live births	82 deaths per 1,000 live births	34 deaths per 1,000 live births	55 deaths per 1,000 live births
	Uganda	48 deaths per 1,000 live births	76 deaths per 1,000 live births	54 deaths per 1,000 live births	66 deaths per 1,000 live births
Measles immunization coverage	India	85 %	40 %	72 %	54 %
	Uganda	65 %	49 %	68 %	55 %
Skilled attendant at delivery	India	74 %	38 %	89 %	19 %
	Uganda	80 %	37 %	76 %	28 %
Antenatal care coverage	India	89 %	69 %	97 %	54 %
	Uganda	97 %	93 %	96 %	93 %

Sources: [27, 28, 30, 31, 34, 47–49]

#### Sub-national level

Once a worst-off country is identified, the next step for high-income country researchers is to identify a research population by examining who the worst-off groups and communities are within it. For LMIC researchers, their first step is to identify a worst-off sub-national population in their country. (Again, this can be based on other LMIC actors’ prior identification of such populations). To identify candidate research populations, researchers (or other LMIC actors) ought to start by considering what individual or community characteristics are often associated with poor health in a given country that should not be so in a just world and/or what social arrangements create and entrench poor health in that country (Table 1). Relevant individual characteristics likely will include living in a certain area (conflict-affected, environmentally fragile, rural, slum, and/or remote), poverty, race, ethnicity, being a single mother, being a refugee or asylum seeker, membership in stigmatised group, having a disability, and working in the informal sector. Relevant community characteristics likely will include those associated with a lack of community capability for health and health system development such as poor social cohesion (ability to work together to solve community health problems), limited participatory decision-making on health matters, and a lack of physical and financial resources for health systems [9, 20]. Many of the above characteristics (and some social arrangements) will be associated with poor health in many worst-off countries, but there are also likely to be country-specific characteristics and social arrangements associated with poor health. Some overlap will occur between groups identified by shared characteristics and experience of social arrangements and practices. For example, groups identified based on their shared experience of stigmatization may be a particular race or ethnicity.

Researchers, preferably in collaboration with in-country stakeholders, should select one or more of these characteristics or social arrangements and determine whether or not a sizeable gap actually exists between the level of health achieved by the sub-national population with the characteristic(s), or who is affected by the social arrangement, and the level of health achieved by the relevant comparator sub-national population in the given country. Beyond looking at the sub-national population's achieved health in terms of health outcomes, it will also be useful to rely on burden of disease indicators when conducting biomedical studies and to rely on health system indicators when conducting HSR. This is because such indictors show who is worst-off in ways that the research type is capable of helping to combat. Clinical research can help address a high burden of disease by developing new medical products; HSR can help address poor access to health services and financial protection by developing, for example, new service delivery models or health insurance schemes.

Where existing data indicates that there is a substantial difference in health outcomes between, for example, the lowest and highest income quintiles and between populations living in slum and non-slum urban areas, this would support the conduct of research on poor populations, slum-dwelling populations, or poor *and* slum-dwelling populations within a given worst-off country. A number of metrics exist that can be used to measure health inequalities between groups or communities. Some can quantify differences between socioeconomic status groupings, whereas others measure differences between population groupings by race, gender, and geographic location [29]. All metrics have limitations and, therefore, it is recommended that more than one measure be employed [29].

To further explore what taking this approach entails in practice, it is used to assess whether or not the research populations in the HSR cases (see “Cases of international health research projects,” above) were worst-off in terms of their health. In the Ugandan project, three eastern rural districts (Kamuli, Kibuku, Pallisa) were selected in 2011. The relevant comparison group in that case was the urban population in Uganda. Available evidence shows that health outcomes and access to health services did vary between these populations in 2011 (Table 3) [30]. This evidence supports classifying the rural population within Uganda as a worst-off population. Even so, it is important to note that 80 % of the Ugandan population was rural in 2011 [31]. As a result, there was considerable variation within the rural population, with certain rural districts’ health achievements falling quite close to those of urban districts. This raises the question: is it necessary to choose to work in rural districts with more sizeable gaps in health status from urban districts? For instance, districts in northern Uganda generally perform worse than districts in other regions and, within the north, the *Karamoja region* performs much worse in terms of health outcomes and health system performance than other districts [32, 33]. In response, it is again suggested that, while researchers are not required to conduct studies in the *absolute* worst-off rural districts, they should at a minimum select rural districts with a *substantial* gap in health or access to services compared to the urban population. The districts should fall within the bottom third of all districts in terms of health outcomes and health system performance.

In Uganda, there is limited publically available data on health outcomes in different districts. However, some information (in the form of District League Tables) does exist that can help identify rural districts with poor health system performance, though these tables do not measure access beyond a few services and do not measure financial protection (Table 4). If rankings solely from 2011 are used, none of the three districts were amongst the worst-off because they did not score in the bottom third of all districts in Uganda. If performance over time were considered, Kamuli was clearly worst-off in terms of its health system performance between 2005 and 2010, but it improved considerably since then. In 2011, it was amongst the top third performing districts in Uganda.

**Table 4:** Health system performance rankings of Kamuli, Kibuku, and Pallisa districts 2005-2011

	2004/05 MOH	2006/07 MOH	2008/09 MOH	2009/10 MOH	2010 MOH	2010/2011 MOH	2011 MOH
Kamuli	46^th^ or below (in bottom 10 districts)	55^th^ or below (in bottom 15 districts)	62^nd^	74^th^	76^th^	29^th^	29^th^
Kibuku	NA	NA	NA	NA	NA	70^th^	70^th^
Pallisa	Between 11^th^ and 45^th^	Between 16^th^ and 54^th^	21^st^	58^th^	58^th^	26^th^	26^th^
Total Number of Districts	56^th^	80^th^	80^th^	80^th^	80^th^	111^th^	111^th^

Note: MOH indicates that the data comes from the Ugandan Ministry of Health. Kibuku district was established in 2010/2011. Sources: [32, 50–55]

In the HSR case from India, the Sundarbans’ population was selected to be the focus of the study. That population has two traits associated with poor health: extreme poverty and living in an area that is vulnerable to climactic shocks. The relevant comparator sub-national populations for the Sundarbans’ population are affluent populations in India, populations living in environmentally-secure areas of India, and affluent populations living in environmentally-secure parts of India. Unfortunately, available data at the time of the project—namely, the National Family Health Survey in India (2005–06)—was not disaggregated by living in an environmentally secure or insecure area. It did demonstrate a substantial gap between rich and poor populations within India in terms of health outcomes and access of health services [34]. As a poor population within India, the inhabitants of the Sundarbans could, therefore, potentially be considered worst-off in terms of their health.

Existing data on access to health services showed that 29 % of deliveries occurred in a health facility in the Sundarbans compared to 84 % of deliveries amongst the highest income quintile and 58 % of deliveries amongst the lowest income quintile in India. In West Bengal state, the delivery rate at health facilities was 43 %. In India as a whole, it was 39 % and, in Kerala state, it was 99 % [34, 35]. The quality of health care was likely to be low in the Sundarbans because 62 % of outpatients went to Rural Medical Practitioners (who practice medicine without formal training) due to physical proximity and cost. This was compared to 54 % of outpatients in rural West Bengal [28]. Overall, the data available was consistent with classifying the Sundarbans’ population as among the worst-off in health terms in 2011.

### Systematic disadvantage

#### Two measures of poverty

Where the worst-off are defined in terms of individual systematic disadvantage, external researchers from high-income countries should perform HSR in countries with substantial shortfalls on three or more dimensions of well-being, including health. Identifying which countries’ populations are systematically disadvantaged would ideally entail measuring the dimensions of well-being identified by philosophers using metrics that can be compared across countries. This task is very complex to do at the national level let alone the sub-national level. Thus far, metrics have not been developed, though some work has been done towards that aim by Wolff and de-Shalit [10].

As an alternative, an approach is proposed that draws on Powers and Faden’s theory of social justice. This theory uses proxies for individual systematic disadvantage that are helpful to rely upon to identify worst-off populations at the national and sub-national levels: domination linked to group membership and poverty. Patterns of systematic disadvantage flow from disparities in resources and disparities in respect. Group domination is characterised by the central moral evil of reduced respect. Group membership “becomes sufficient reason for failing to treat people as dignified human beings worthy of equal moral concern” ([11], p.87). Dominated groups can be defined by different characteristics such as gender, ethnicity, race, caste, sexual orientation, and/or disability [11]. Systematic disadvantage also flows from “dramatic differences in material resources [that] produce a cluster of deficiencies in well-being that makes it extremely unlikely that individuals can improve their life prospects through their own efforts” ([11], p. 90). No inequalities are more pressing for public health to confront than those associated with severe, life-long poverty in LMICs [11].

Relying upon the theory’s proxy indicators, priority would be afforded to countries and research populations that experience the greatest poverty or domination due to group membership (Table 1). According to Alkire and Santos,

*[t]here are essentially two methods to measure poverty, the direct method and the indirect or income approach. The direct method shows whether people satisfy a set of specified basic needs, rights, or – in line with Sen’s capability approach – functionings. The indirect method determines whether people’s incomes fall below the poverty line – the income level at which some specified basic needs can be satisfied* ([36], p. 5).

While recognising that other metrics of poverty exist, below the poverty line measures and the multidimensional poverty index are introduced and applied below (such cases may also be built using other metrics). This choice reflects the fact that they comprise commonly used metrics for directly and indirectly measuring poverty respectively. They also reflect two different conceptions of poverty, which may have implications for who is identified as worst-off and is, therefore, important to explore. Significant data has been collected using these metrics that can be relied upon to assess the two cases.

At a national level, the indirect or income approach assesses countries’ GDP or GNI per capita. The World Bank classification of countries as low, lower middle, upper middle, and high-income countries relies on such an approach [37]. At the sub-national level, the indirect approach considers what proportion of individuals in different groups or communities fall below the poverty line (BPL) in their country. The poverty line is the minimum level of income deemed adequate to live on in a given country. Where the indirect approach is employed, factors that bear consideration when determining whether a country’s population is systematically disadvantaged include its income level classification; the length of time the country has been classified as such; and its level of within-country income inequality. At the sub-national level, priority is afforded to dominated groups and the poorest within countries. Using BPL measures to identify the poorest within countries could entail identifying where: 1) a group or community falls in the bottom third of performers within the country (in terms of the proportion of its members living BPL) or 2) a substantially higher proportion of the group or community lives BPL relative to the group or community with the lowest proportion of individuals living BPL in the country.

As an alternative to income-based poverty metrics, the Multidimensional Poverty Index (MPI) has been utilised to measure poverty within 110 LMICs. The MPI assumes that poverty is a condition in which people are exposed to multiple disadvantages, which is consistent with Wolff and de-Shalit’s concept of corrosive disadvantage and Powers and Faden’s concept of systematic disadvantage [38]. Multidimensional poverty measurement focuses on a set of ten deprivations across three dimensions—health, education, and standard of living—related to the Millennium Development Goals [36]. The ten indicators are: years of schooling, school attendance, child mortality, nutrition, electricity, sanitation, water, floor, cooking fuel, and assets. Each dimension is equally weighted and each indicator within a dimension is also equally weighted [39]. A person is identified as multidimensionally poor if s/he is deprived in at least one third of the ten indicators. If a person is deprived in 20–33.3 % of the ten indicators, s/he is considered ‘Vulnerable to Poverty’, and if s/he is deprived in 50 % or more s/he is identified as being in ‘Severe Poverty’ [39].

The MPI serves to identify people with “joint” disadvantages [38]. However, it only includes three potential dimensions of poverty because comparable data of sufficient quality are not available from the public domain for 100+ LMICs to consider any other dimensions [36]. As a result, the extent to which the MPI dimensions overlap with the broad dimensions of well-being identified by philosophers and its ten indicators’ capacity to measure those dimensions is not ideal. For example, if the *six* dimensions of well-being identified by Powers and Faden are considered, the MPI dimensions of health, education, and standard of living can be encompassed within the dimensions of health, reasoning, and self-determination respectively.

Beyond poverty and group domination, another potential proxy for individual systematic disadvantage at the sub-national level is a lack of community capability across sectors (i.e. not just the health sector). Some community capability metrics have been developed that could be adapted for that purpose, though they would need to vary and be validated by country [20, 40]. Due to a lack of existing data, this approach is not applied below.

#### Applying the income approach

Relying on the income approach to identify the worst-off excludes countries with high levels of wealth even if they have poor health outcomes. Turning to the two cases, the World Bank classifies Uganda as a low-income country and it is, therefore, considered extremely poor. At the sub-national level, however, it is less clear whether Pallisa, Kibuku, and Kamuli were some of the poorest districts within the country in 2011. In Uganda, the best performing districts had only 7.7 % and 13.06 % of their populations falling BPL. The worst performing districts were primarily in the northern region, with 67–89 % of their populations falling BPL. In Pallisa, the figure was 53 % and it was 49 % for both Kibuku and Kamuli. These three districts exhibited a substantial gap from the best performing districts in Uganda [41]. Yet, Pallisa, Kamuli, and Kibuku also exhibited a gap from the worst performing districts and did not fall in the bottom third of districts in Uganda. As a result, they were not considered systematically disadvantaged by this approach.

Since the World Bank classifies India as a lower middle-income country, it is not automatically considered extremely poor. Over time, India’s average wealth has been rising. In terms of in-country income inequality, India’s Gini coefficient (33.9) is not so high compared to other “emerging economies” such as South Africa (63.1), Brazil (54.7), and China (42.1). However, in India, the ratio between the top and the bottom wage-earners has doubled since the early 1990s [42]. In Brazil and South Africa, the ratio was almost halved between the early 1990s and late 2000s. Whether India could be classified as systematically disadvantaged in 2011 is then somewhat ambiguous. If lower middle-income countries are not classified as extremely poor, it comes down to whether India’s expanding income inequality was sufficient for it to be considered worst-off. Given that India’s income inequality was not as large as other middle-income countries, it either was not worst-off or, at most, would have been a low priority for external researchers performing HSR in LMICs. Alternatively, if both low-income and lower middle-income countries are classified as extremely poor, then India was considered worst-off.

In the latter case, the next step would be to assess whether the Sundarbans’ population was one of the poorest in India in 2011. Over 40 % of households in the Sundarbans were BPL and 13 % were classified as the poorest of the poor [35]. This means that the Sundarbans matched the *worst* performing state in all of India in terms of the proportion of its population falling BPL. In 2011, 39.9 % of Chhattisgarh state’s population lived BPL. Comparatively, 20 % of West Bengal’s population lived BPL [43]. Additionally, 46 % of the Sundarbans’ population belonged to traditionally marginalised groups in India [35]. Thus, the Sundarbans were considered among the worst-off within India.

#### Applying the MPI approach

India and Uganda each fell considerably short of the optimal MPI achieved worldwide (i.e. 0) (Table 5). They fell into the bottom third of the 110 LMICs for which multidimensional poverty has been measured for both their MPI score and their inequality amongst the poor score (Table 5). The former measures the proportion of the population that is multidimensionally poor and the latter measures inequality in deprivation counts among the poor and disparities across groups [39]. The MPI approach, therefore, identified India and Uganda as among the worst-off.

**Table 5:** Multidimensional poverty in India and Uganda

	India	Uganda
Multidimensional poverty index	0.283	0.367
MPI Ranking (of 110 countries)	82^nd^	96^th^
Percentage of poor people	53.7 %	69.9 %
Vulnerable to poverty	16.4 %	19.0 %
In severe poverty	28.6 %	38.2 %
Inequality among the MPI poor	0.234	0.192
Inequality Ranking (of 110 countries)	97^th^	84^th^

Sources: [39, 44]

At the sub-national level, MPI data exists for states in India and for regions in Uganda. In India, the range of states’ percentage of multidimensionally poor people was 12.7 % (Kerala) to 79.3 % (Bihar) [39]. West Bengal, the state where the Sundarbans are located, exhibited a sizeable gap relative to the best performing state and fell in the bottom third of states on both indicators (9^th^ of 29 states), with 57.4 % of its population classified as multidimensionally poor [39]. As such, West Bengal’s population, including the Sundarbans, would have been considered worst-off within India.

In Uganda, the range of regions’ percentage of multidimensionally poor people was 17.3 % (Kampala) to 96.5 % (Karamoja) [44]. The two Eastern regions, where Kamuli, Kibuku, and Pallisa are located, fell third and fifth on both indicators, with 81.7 % and 74.7 % of their populations classified as multidimensionally poor respectively [44]. Thus, all three districts are within regions whose populations’ experience substantial shortfalls from the lowest level of multidimensional poverty achieved in Uganda (17.3 %). Those district(s) in the East Central region did not fall into the bottom third of performers by region. However, as all regions of Uganda except for Kampala had 60 % or more of their populations classified as multidimensionally poor, it is argued that these districts were amongst the worst-off. Having 74.7 % of their populations classified as multidimensionally poor is sufficiently close to the worst performing regions to have been considered worst-off.

## Conclusions

Ethical guidance consistent with egalitarian theories of social justice has been developed that calls for health systems researchers (from high-income countries and LMICs) to focus on the worst-off. In this paper, we sought to operationalise that guidance and provide researchers, including those performing studies to unlock community capabilities to improve local health systems, with a methodology for identifying worst-off populations. We identified two broad definitions of worst-off; they can be understood as either those who are least advantaged on multiple dimensions of well-being including health *or* those who are least advantaged in terms of their health. How these two concepts can be used to select worst-off countries and research populations was described.

In applying the concepts to real-world HSR cases, we also sought to explore whether relying on them results in uniform or varying classifications of countries and research populations (Table 6). The cases indicate the two concepts can identify different countries as appropriate places for external researchers from high-income countries to conduct HSR, based on their relying on income, MPI, or health achievements to classify countries as worst-off. While Uganda was classified as worst-off by both concepts, it can be argued that India should be excluded under the income approach because it is now a middle-income country.

**Table 6:** Classifications at national and sub-national levels according to three metrics of worst-off

	Worst-off in terms of health	Systematic disadvantage- BPL approach	Systematic disadvantage- MPI approach
India	Yes	Maybe	Yes
Sundarbans	Yes	Yes	Yes
Uganda	Yes	Yes	Yes
Kamuli, Kibuku, Pallisa districts	No	No	Yes

At the sub-national level, there can be a high degree of overlap when using the two concepts to identify appropriate groups or communities upon whom to focus HSR (Table 6). This will occur when sub-national populations that are worst-off in health terms are identified using either of the following characteristics—being a member of a dominated group or being poor within a country. The Sundarbans’ population, for example, is extremely poor and was classified as being worst-off using both concepts. However, there will be less overlap between the two approaches where characteristics associated with poor health are used to select research populations *and* those characteristic(s) aren’t strongly associated with poverty (BPL or MPI) or membership in a dominated group in the given country. As the Uganda case shows, deficits in non-health dimensions of well-being can also lead sub-populations that are not worst-off in health to be classified as systematically disadvantaged (Table 6).

This exploration demonstrates that the decision to use one concept of worst-off as opposed to the other does matter. We do not take a position as to which of the two concepts should be used, though perhaps more convergence exists in the philosophical literature around the systematic disadvantage approach [45]. Clusters of disadvantage bear greater moral weight than deficits in a single dimension of well-being and are, therefore, more urgent to address. However, debate over the two concepts of disadvantage continues. Thus, we recommend that health researchers (and other actors) should use the concept that best reflects their moral commitments—namely, to perform research focused on reducing health inequalities or systematic disadvantage more broadly. If they want to address the latter, we further suggest relying on the MPI approach because the income approach is less likely to identify countries or sub-national populations with substantial health deficits as worst-off. (The MPI approach incorporates health indicators).

Given the current state of data available, the authors again emphasise that the classifications made in this paper are provisional at best. Future work might further examine the link between the concept of worst-off and community capability, e.g. what it means to define the worst-off at the community level (in terms of health and in terms of well-being) rather than the individual level. It might consider when it is ethically acceptable to perform HSR with populations that are not worst-off, identifying what competing obligations or considerations might preclude working with the worst-off in a given country. While emphasising the need for more conceptual work, debates, and discussions, this preliminary guidance can, nonetheless, be of use to health systems researchers aiming to work with and empower disadvantaged groups and communities in LMICs.

## Abbreviations

BPL: Below the poverty line
GDP: Gross domestic product
GNI: Gross national income
HSR: Health systems research
LMICs: Low and middle-income countries
MPI: Multidimensional poverty index
TRIPS: Trade-Related Aspects of Intellectual Property Rights
